# Machine Learning for Predicting Micro- and Macrovascular Complications in Individuals With Prediabetes or Diabetes: Retrospective Cohort Study

**DOI:** 10.2196/42181

**Published:** 2023-02-27

**Authors:** Simon Schallmoser, Thomas Zueger, Mathias Kraus, Maytal Saar-Tsechansky, Christoph Stettler, Stefan Feuerriegel

**Affiliations:** 1 Institute of AI in Management LMU Munich Munich Germany; 2 Munich Center for Machine Learning (MCML) Munich Germany; 3 Department of Diabetes, Endocrinology, Nutritional Medicine and Metabolism Inselspital Bern University of Bern Bern Switzerland; 4 Department of Endocrinology and Metabolic Diseases Kantonsspital Olten Olten Switzerland; 5 Institute of Information Systems FAU Erlangen-Nuremberg Nuremberg Germany; 6 The McCombs School of Business The University of Texas at Austin Austin, TX United States

**Keywords:** diabetes, prediabetes, machine learning, microvascular complications, macrovascular complications

## Abstract

**Background:**

Micro- and macrovascular complications are a major burden for individuals with diabetes and can already arise in a prediabetic state. To allocate effective treatments and to possibly prevent these complications, identification of those at risk is essential.

**Objective:**

This study aimed to build machine learning (ML) models that predict the risk of developing a micro- or macrovascular complication in individuals with prediabetes or diabetes.

**Methods:**

In this study, we used electronic health records from Israel that contain information about demographics, biomarkers, medications, and disease codes; span from 2003 to 2013; and were queried to identify individuals with prediabetes or diabetes in 2008. Subsequently, we aimed to predict which of these individuals developed a micro- or macrovascular complication within the next 5 years. We included 3 microvascular complications: retinopathy, nephropathy, and neuropathy. In addition, we considered 3 macrovascular complications: peripheral vascular disease (PVD), cerebrovascular disease (CeVD), and cardiovascular disease (CVD). Complications were identified via disease codes, and, for nephropathy, the estimated glomerular filtration rate and albuminuria were considered additionally. Inclusion criteria were complete information on age and sex and on disease codes (or measurements of estimated glomerular filtration rate and albuminuria for nephropathy) until 2013 to account for patient dropout. Exclusion criteria for predicting a complication were diagnosis of this specific complication before or in 2008. In total, 105 predictors from demographics, biomarkers, medications, and disease codes were used to build the ML models. We compared 2 ML models: logistic regression and gradient-boosted decision trees (GBDTs). To explain the predictions of the GBDTs, we calculated Shapley additive explanations values.

**Results:**

Overall, 13,904 and 4259 individuals with prediabetes and diabetes, respectively, were identified in our underlying data set. For individuals with prediabetes, the areas under the receiver operating characteristic curve for logistic regression and GBDTs were, respectively, 0.657 and 0.681 (retinopathy), 0.807 and 0.815 (nephropathy), 0.727 and 0.706 (neuropathy), 0.730 and 0.727 (PVD), 0.687 and 0.693 (CeVD), and 0.707 and 0.705 (CVD); for individuals with diabetes, the areas under the receiver operating characteristic curve were, respectively, 0.673 and 0.726 (retinopathy), 0.763 and 0.775 (nephropathy), 0.745 and 0.771 (neuropathy), 0.698 and 0.715 (PVD), 0.651 and 0.646 (CeVD), and 0.686 and 0.680 (CVD). Overall, the prediction performance is comparable for logistic regression and GBDTs. The Shapley additive explanations values showed that increased levels of blood glucose, glycated hemoglobin, and serum creatinine are risk factors for microvascular complications. Age and hypertension were associated with an elevated risk for macrovascular complications.

**Conclusions:**

Our ML models allow for an identification of individuals with prediabetes or diabetes who are at increased risk of developing micro- or macrovascular complications. The prediction performance varied across complications and target populations but was in an acceptable range for most prediction tasks.

## Introduction

### Background

Micro- and macrovascular complications are a major burden for individuals with diabetes, resulting in an increased risk of morbidity and mortality [1]; for example, individuals with diabetes are at increased risk of developing cardiovascular disease (CVD) in comparison with individuals without diabetes [2]. Furthermore, macrovascular complications are responsible for the majority of diabetes-related deaths [3]. Other examples are diabetic retinopathy, which is the primary cause of blindness among adults aged 20 to 74 years [4], and diabetic nephropathy, which is responsible for the majority of new cases of renal failure in the United States [5]. Furthermore, it has been shown that already individuals with prediabetes have an increased risk of developing micro- or macrovascular complications [6,7].

Hence, identifying these individuals (with either prediabetes or diabetes) at increased risk of developing micro- or macrovascular complications is important to allocate treatments, which might prevent the onset of these complications. Importantly, interventions have proven useful in reducing the risk of developing micro- or macrovascular complications in individuals with diabetes [8,9], and targeted interventions to those at highest risk were shown to be more effective than population-wide interventions [10]. Although several risk factors for the different micro- and macrovascular complications are known, identifying those at highest risk is complex, and machine learning (ML) may improve the prediction performance.

### Objectives

Prior studies have used traditional statistical methods (eg, Cox proportional hazards models) to predict complications in individuals with diabetes [11,12]. Such methods typically have a linear structure and thus have the advantage of being interpretable. However, they usually cannot effectively handle high-dimensional data. By contrast, ML allows for modeling more complex (eg, nonlinear) relationships between predictors and outcomes and thus makes effective use of high-dimensional data as in the case of electronic health records (EHRs). Therefore, in recent studies, ML methods were increasingly applied for predicting complications in individuals with diabetes [13-18]. However, these studies suffer from (1) a small and nonrepresentative population (eg, individuals in a single hospital) or (2) a limited number of complications, or (3) they lack important patient information (eg, biomarkers or disease codes). Furthermore, according to a review of prediction models for diabetes complications [19], there exist no prediction models for micro- or macrovascular complications in individuals with prediabetes. However, such prediction models are important because they allow for an earlier intervention (ie, treatment via medications or lifestyle changes) to prevent the onset of micro- or macrovascular complications. Therefore, we aimed to develop ML models (logistic regression and gradient-boosted decision trees [GBDTs]) for predicting micro- and macrovascular complications in individuals with diabetes or prediabetes over a forecast horizon of 5 years.

## Methods

This section was structured in accordance with the Transparent Reporting of a Multivariable Prediction Model for Individual Prognosis or Diagnosis (TRIPOD) statement for the development of prediction models in medicine [20].

### Source of Data

This is a retrospective analysis, where we analyzed anonymized EHRs from an Israeli health provider [21,22]. The EHRs contain data from multiple centers across Israel and cover the years from 2003 to 2013. The EHRs consist of 6 tables in a longitudinal data format with information on demographics (age and sex), blood pressure, BMI, biomarkers, medications, and disease codes. The baseline year to build the prediction models was 2008. We used a 5‑year forecast horizon, that is, the end of follow-up was in 2013. This forecast horizon was chosen analogous to previous work [11,16-18] because it allows for identification of the individuals at highest risk for developing a micro- or macrovascular complication.

### Participants

We built our prediction models for 2 different target populations, consisting of individuals with either prediabetes or diabetes. Definitions of prediabetes and diabetes were based on laboratory measurements of glycated hemoglobin (HbA_1c_), recorded disease codes (using the International Classification of Diseases, Ninth Revision [ICD‑9]), and medications. Onset of diabetes was defined by (1) 2 measurements of HbA_1c_ ≥6.5% (48 mmol/mol), where the onset is then set to the year of the first measurement; (2) an ICD‑9 code corresponding to diabetes (249 or 250); or (3) if any prescription for antidiabetic medication or device for self-measurement of blood glucose was recorded. The list of antidiabetic medications and devices for self-measurement of blood glucose is presented in Multimedia Appendix 1. An individual was considered to have prediabetes if either a single measurement of HbA_1c_ of 5.7% to 6.4% (39 mmol/mol-47 mmol/mol) or an ICD‑9 code corresponding to prediabetes (790.2) was recorded. In addition, individuals with prediabetes were only considered if none of the aforementioned definitions of diabetes were fulfilled.

**Figure 1:**
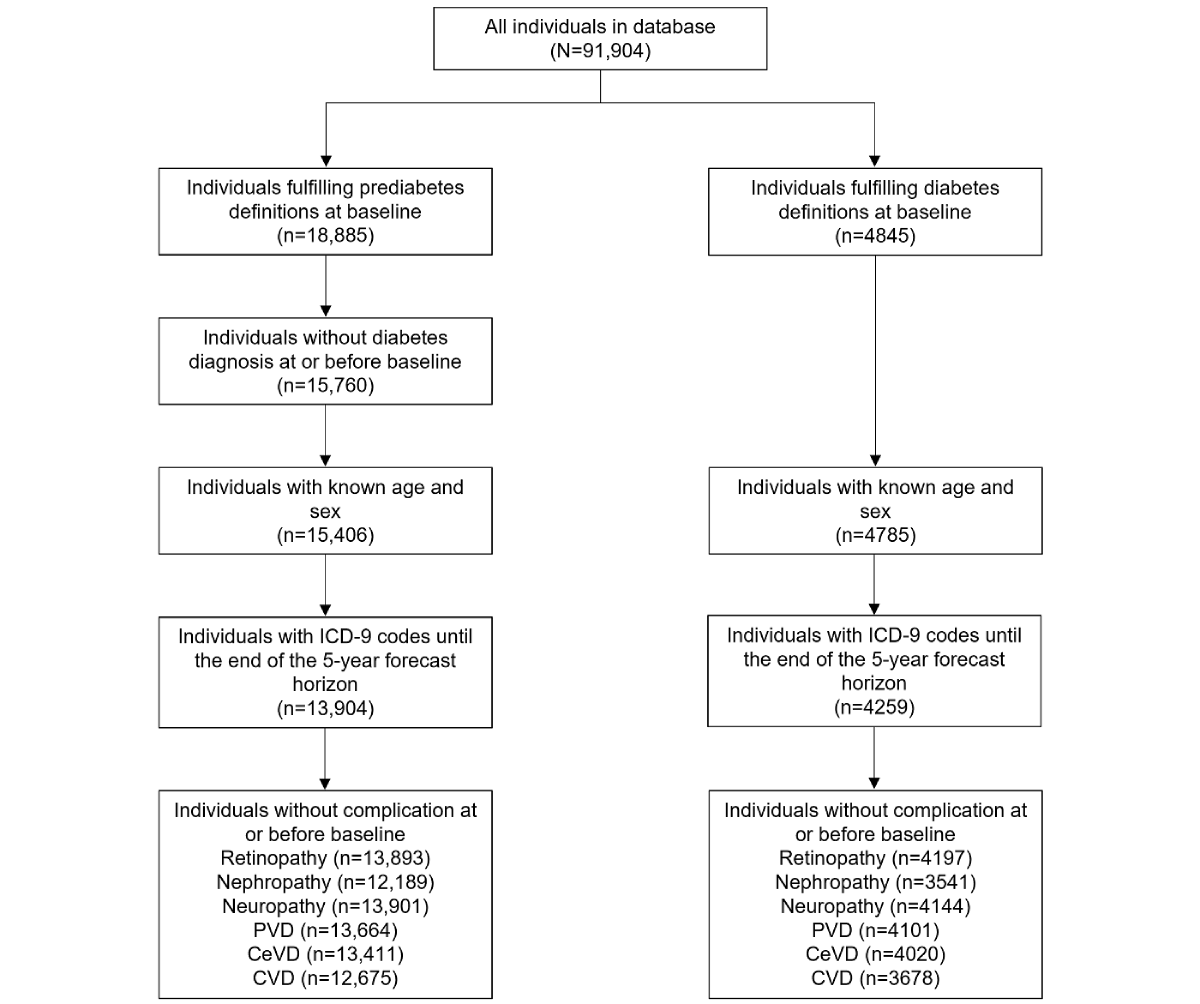
Flowchart of the inclusion criteria. CeVD: cerebrovascular disease; CVD: cardiovascular disease; ICD-9: International Classification of Diseases, Ninth Revision; PVD: peripheral vascular disease.

Inclusion and exclusion criteria were as follows: we selected individuals who were considered to have prediabetes or diabetes in 2008. We only included individuals where information on sex and age was recorded. To account for patient dropout, we only considered individuals where ICD‑9 codes were recorded until 2013. This ensured that, for each individual, an ICD‑9 code corresponding to a possible complication could have been recorded over the entire 5‑year forecast horizon. For nephropathy, we required that either ICD‑9 codes or measurements of serum creatinine or the albumin-to-creatinine ratio in the urine were recorded until 2013. In addition, we only included individuals at baseline who had not been diagnosed with the specific complication beforehand. Hence, the baseline populations differed across complications. A flowchart of the inclusion and exclusion criteria is displayed in Figure 1.

### Outcome

Definitions of micro- and macrovascular complications were based on recorded ICD‑9 codes. We included the following microvascular complications: retinopathy (ICD‑9 codes 250.5 and 362.0), nephropathy (ICD‑9 codes 250.4 and 585), and neuropathy (ICD‑9 codes 250.6 and 357.2). Furthermore, we considered 3 macrovascular complications: peripheral vascular disease (PVD; ICD‑9 codes 250.7, 443.9, and 440), cerebrovascular disease (CeVD; ICD‑9 codes 430, 431, 432, 433, 434, 435, 437, and 438), and CVD (ICD‑9 codes 410, 411, 412, 413, and 414).

For nephropathy, we additionally included the ratio of albumin to creatinine in the urine (albuminuria) and the estimated glomerular filtration rate (eGFR) as disease-defining markers. We considered an individual to have developed nephropathy if 1 measurement of eGFR <60 ml/minute per 1.73 m^2^ or 2 measurements of the ratio of albumin to creatinine in the urine ≥30 mg/g were recorded [23]. The eGFR has been calculated using the formula from the study by Levey et al [24], which takes serum creatinine, age, sex, and ethnicity as inputs. As the formula only differentiates between a Black ethnicity and a non‑Black ethnicity, we assumed a non-Black ethnicity for all individuals because the EHRs did not cover that information but represent an Israeli population.

### Predictors

The following predictors were used: age, sex, BMI, and blood pressure (systolic blood pressure [SBP] and diastolic blood pressure). We further included predictors from the following categories: biomarkers, medications, and ICD‑9 codes. Feature selection was applied, whereby we selected the most frequent biomarkers, medications, and ICD‑9 codes. Specifically, we added to our predictors the 60 most frequently recorded biomarkers and the 20 most frequently recorded ICD‑9 codes. We did not include the ICD‑9 code 250 (diabetes mellitus) because this code was used in our inclusion criteria to define the diabetes cohort (among other criteria). We grouped the 50 most often prescribed medications into 20 classes (Multimedia Appendix 1) and added them to our predictors. In total, this resulted in 105 predictors (Multimedia Appendix 2).

The predictors were preprocessed by applying the following steps: first, ICD‑9 codes and medications were one-hot encoded, and ICD-9 codes were forward filled to account for the disease history of the individual. Second, we averaged measurements for BMI, blood pressure, and biomarkers if multiple measurements were recorded within 1 year. Third, missing values at the point of evaluation were forward filled from previous years. For the logistic regression, measurements that were still missing were imputed using the median. For the GBDTs, we did not impute missing data because the chosen model can handle missing values automatically. Finally, the predictors were standardized (by removing the mean and dividing by the SD) for the logistic regression. By contrast, standardization is not necessary for the GBDTs.

### Sample Size

The primary end point of this study was the diagnostic accuracy of our ML models to predict micro- and macrovascular complications. Therefore, we included all individuals who fulfilled our inclusion criteria (as specified in the Participants subsection) to maximize the discriminatory power of our models.

### Missing Data

Missing data in the predictors were handled as follows: we only included individuals with recorded age and sex. Hence, no data are missing for these predictors. For numerical values (ie, blood pressure, BMI, and biomarkers), missing values at the baseline were forward filled from the last measurement. For the logistic regression, values that were still missing were imputed using the median. For the GBDTs, no imputation was performed as described previously, but, internally, GBDTs treat missing values as informative and replace them with a dummy variable [25].

### Statistical Analysis Methods

We built separate ML models for each complication and disease state at baseline (either prediabetes or diabetes). Specifically, we used a logistic regression with L1 regularization and GBDTs. Logistic regression is a linear model, which typically performs well in clinical settings [26]. It is inherently interpretable, which is advantageous—and often demanded—for medical predictions [27]. Gradient boosting is an ML technique where a sequence of weak learners (here, decision trees) are sequentially optimized to minimize the prediction errors of the previous weak learners. The final gradient-boosting model consists of an ensemble of weak learners. GBDTs are highly effective in modeling complex, nonlinear relationships and in handling high-dimensional data as in the case of EHRs. Both models were chosen because they are well-established in the medical literature [26,28,29]. Furthermore, this allows us to make direct comparisons between a linear model (logistic regression) and a more flexible model (GBDTs).

The ML models were implemented in Python (version 3.6.9; Python Software Foundation). In particular, we used *scikit‑learn* (version 0.23.2 [30]) for the logistic regression and the CatBoost package (version 1.0.4 [25]) for the GBDTs.

We applied a nested cross-validation, where we used 5 outer folds to measure the out‑of‑sample performance of the ML models and 4 inner folds to choose the optimal hyperparameters (Multimedia Appendix 3). More specifically, within each training set in the outer fold, an additional 4-fold cross-validation is performed to select the hyperparameters. Thereafter, the model is trained on the training set using these optimal hyperparameters, and the out-of-sample performance is evaluated on the test set corresponding to the current outer fold. This procedure is repeated within each of the 5 folds of the outer cross-validation. As such, nested cross-validation is best practice in ML to optimize the hyperparameter tuning and to assess how well the model generalizes to new data because it ensures that each individual in the data set is used once for measuring the out-of-sample performance [31].

We evaluated the performance of our ML models primarily on the area under the receiver operating characteristic curve (AUROC). We report the mean and the SD of the AUROC across the 5 different test sets generated by the outer cross-validation. For discussing the results, we categorized the AUROC into moderate (0.600-0.700), acceptable (0.700-0.800), and good (0.800-0.900). Additional performance metrics such as area under the precision recall curve, sensitivity, specificity, and balanced accuracy are reported in Multimedia Appendix 4.

The calibration (observed risk vs raw prediction score) of a prediction model is often relevant in medical settings [32]. In contrast to logistic regression, GBDTs may not be well calibrated. Therefore, we applied a post hoc calibration by fitting a logistic regression to the predictions on the validation set. We evaluate the calibration in Multimedia Appendix 5 by plotting the calibration curves and reporting the Brier score [33].

To explain the predictions of the GBDTs, we calculated Shapley additive explanations (SHAP) values [34]. These represent a unified approach for estimating the individual contribution of a predictor to the overall model output. Thus, SHAP values provide a ranking of the most important predictors [34]. Furthermore, SHAP values inform whether larger (smaller) values of a predictor are attributed with an increased risk of developing a certain complication. In addition, we report the coefficients of the logistic regression in Multimedia Appendix 6.

### Risk Groups

We followed best practice to check for potential algorithmic bias [35] and thus added a separate analysis, where we evaluated the performance differences between male and female individuals. For this, we did not train new prediction models on only a male or female population but instead checked the performance of our final models on these subgroups. The results are presented in Multimedia Appendix 7.

### Ethics Approval

This study was approved by the ethics committee of the faculty of mathematics, computer science, and statistics at Ludwig Maximilian University Munich (EK-MIS-2022-116). We report the Minimum Information About Clinical Artificial Intelligence Modeling (MI-CLAIM) checklist [36], which was developed to improve transparent reporting of ML in medicine, in Multimedia Appendix 8 [36].

## Results

### Participants

The final cohorts consisted of 13,904 individuals with prediabetes and 4259 individuals with diabetes (Figure 1). Of the 13,904 individuals with prediabetes, 2096 (15.1%) developed diabetes within 5 years. Table 1 (individuals with prediabetes) and Table 2 (individuals with diabetes) show the cohort characteristics at baseline in comparison with the characteristics at the time of diagnosis with a certain complication. Across all complications, the 5‑year incidence was smaller for the individuals with prediabetes than for those with diabetes. The cohorts at baseline contained more female individuals than male individuals (diabetes: 56.3% vs 43.7%, respectively; prediabetes: 53.3% vs 46.7%, respectively). For the macrovascular complications PVD and CVD, this ratio turned the other way: more male individuals developed these complications in both cohorts. The mean age of individuals with prediabetes at baseline was 51.2 (SD 8.7) years and that of individuals with diabetes was 52.9 (SD 9.5) years. Furthermore, BMI, SBP, blood glucose, and HbA_1c_ values were higher at the time of diagnosis of a complication than at baseline. This was observed in both cohorts.

**Table 1:** Characteristics of individuals with prediabetes at baseline and at the time point of first manifestation of the corresponding complication (N=13,904).

				Characteristics of individuals at baseline		Retinopathy		Nephropathy		Neuropathy		PVD^a^		CeVD^b^		CVD^c^
Number of individuals, n				13,904		35		753		58		258		462		544
Number of individuals without specific complication at/before baseline, n				N/A^d^		13,893		12,189		13,901		13,664		13,411		12,675
5-year incidence (%)				N/A		0.3		6.2		0.4		1.9		3.4		4.3
**Demographic data**																
	**Sex, n (%)**															
		Female	7412 (53.3)		20 (57.1)		392 (52.1)		29 (50.0)		107 (41.5)		255 (55.2)		188 (34.6)	
		Male	6492 (46.7)		15 (42.9)		361 (47.9)		29 (50.0)		151 (58.5)		207 (44.8)		356 (65.4)	
	Age (years), mean (SD)			51.2 (8.7)		57.3 (8.7)		58.0 (6.8)		57.6 (8.5)		59.8 (6.0)		58.6 (7.2)		57.4 (7.0)
	BMI (kg/m^2^), mean (SD)			30.0 (5.8)		32.0 (5.1)		31.4 (6.1)		33.6 (6.8)		30.2 (5.3)		30.4 (5.0)		30.9 (6.4)
	SBP^e^ (mmHg), mean (SD)			124.8 (15.5)		130.3 (17.0)		128.9 (14.0)		127.5 (14.0)		128.7 (13.5)		128.4 (13.1)		128.5 (13.1)
	DBP^f^ (mmHg), mean (SD)			77.8 (10.9)		80.0 (6.9)		78.8 (7.5)		76.8 (7.7)		78.7 (10.2)		78.4 (7.7)		78.5 (6.9)
**Biomarkers, mean (SD)**																
	Glucose (mg/dL)			97.5 (9.9)		113.51 (14.48)		103.15 (14.12)		111.98 (16.23)		102.46 (11.84)		100.9 (14.11)		100.77 (12.68)
	HbA_1c_^g^ (%)			5.84 (0.26)		6.28 (0.41)		6.05 (0.57)		6.36 (0.59)		6.03 (0.41)		5.97 (0.54)		5.97 (0.46)
	HbA_1c_ (mmol/mol)			40.41 (2.69)		45.16 (4.58)		42.57 (6.17)		45.96 (6.52)		42.39 (4.45)		41.75 (5.81)		41.78 (4.91)

^a^PVD: peripheral vascular disease.

^b^CeVD: cerebrovascular disease.

^c^CVD: cardiovascular disease.

^d^N/A: not applicable.

^e^SBP: systolic blood pressure.

^f^DBP: diastolic blood pressure.

^g^HbA_1c_: glycated hemoglobin.

**Table 2:** Characteristics of individuals with diabetes at baseline and at the time point of first manifestation of the corresponding complication (N=4259).

			Characteristics of individuals at baseline	Retinopathy	Nephropathy	Neuropathy	PVD^a^	CeVD^b^	CVD^c^
Number of individuals, n			4259	59	374	130	123	195	229
Number of individuals without specific complication at/before baseline, n			N/A^d^	4197	3541	4144	4101	4020	3678
5-year incidence (%)			N/A	1.4	10.6	3.1	3.0	4.9	6.2
**Demographic data**									
	**Sex, n (%)**								
		Female	2,397 (56.3)	33 (55.9)	203 (54.3)	68 (52.3)	52 (42.3)	98 (50.3)	96 (41.9)
		Male	1,862 (43.7)	26 (44.1)	171 (45.7)	62 (47.7)	71 (57.7)	97 (49.7)	133 (58.1)
	Age (years), mean (SD)		52.9 (9.5)	58.8 (9.8)	59.7 (6.8)	60.7 (7.4)	61.7 (5.7)	60.2 (7.5)	59.8 (6.5)
	BMI (kg/m^2^), mean (SD)		30.6 (5.8)	32.8 (5.0)	31.9 (5.6)	33.3 (6.3)	32.2 (5.9)	31.1 (5.8)	31.6 (6.5)
	SBP^e^ (mmHg), mean (SD)		126.6 (13.8)	135.0 (16.3)	130.8 (13.5)	128.6 (11.8)	131.6 (12.6)	130.8 (14.7)	129.7 (14.5)
	DBP^f^ (mmHg), mean (SD)		77.7 (7.8)	78.6 (7.4)	78.5 (7.3)	76.6 (5.8)	80.5 (22.5)	78.1 (7.7)	77.8 (6.6)
**Biomarkers, mean (SD)**									
	Glucose (mg/dL)		104.72 (15.54)	123.88 (39.55)	113.15 (22.51)	116.98 (25.79)	114.38 (23.62)	107.38 (14.46)	109.09 (15.07)
	HbA_1c_^g^ (%)		6.16 (0.72)	6.79 (1.0)	6.49 (0.87)	6.59 (0.75)	6.39 (0.79)	6.23 (0.72)	6.41 (0.88)
	HbA_1c_ (mmol/mol)		43.89 (7.93)	50.75 (10.93)	47.42 (9.56)	48.56 (8.21)	46.36 (8.62)	44.65 (7.85)	46.55 (9.55)

^a^PVD: peripheral vascular disease.

^b^CeVD: cerebrovascular disease.

^c^CVD: cardiovascular disease.

^d^N/A: not applicable.

^e^SBP: systolic blood pressure.

^f^DBP: diastolic blood pressure.

^g^HbA_1c_: glycated hemoglobin.

### Prediction Performance

**Figure 2:**
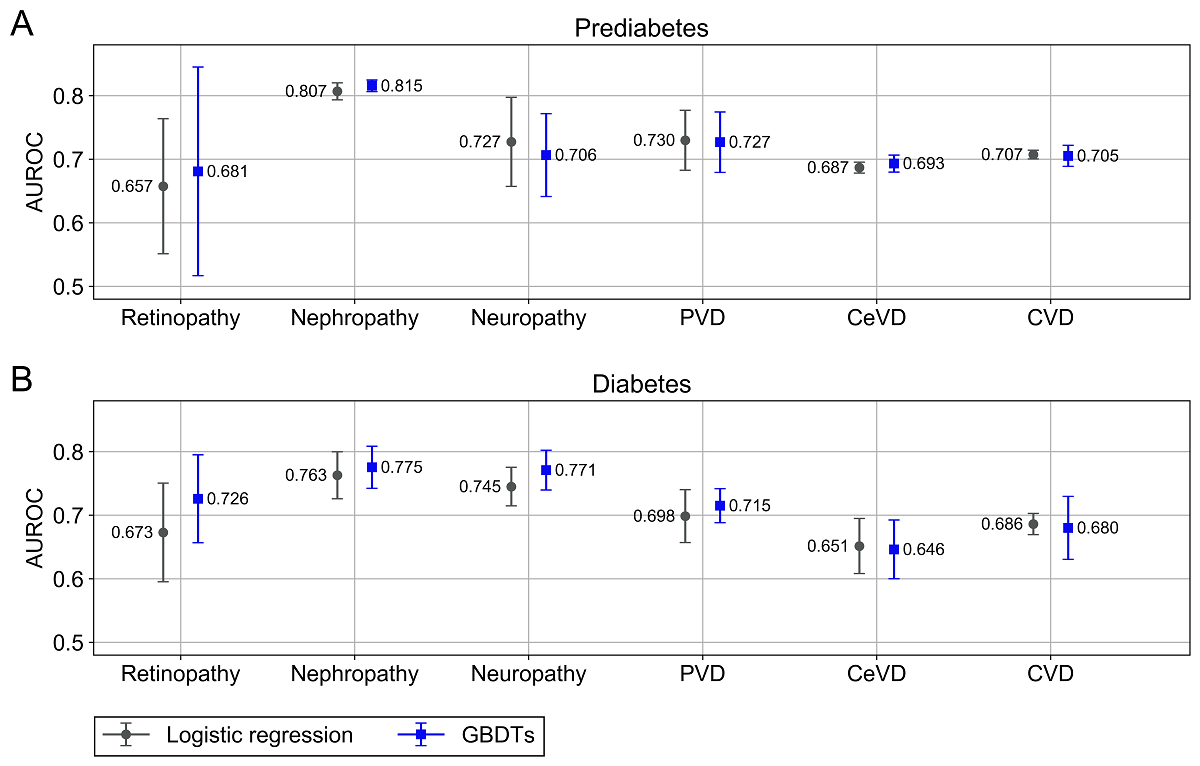
Performance of the logistic regression and the gradient boosted decision trees (GBDTs) for predicting micro- and macrovascular complications in (A) individuals with prediabetes or (B) diabetes. We report the mean of the area under the receiver operating characteristic curve (AUROC) across the 5 different test sets. The error bars denote SD. CeVD: cerebrovascular disease; CVD: cardiovascular disease; PVD: peripheral vascular disease.

Figure 2 shows the performance of the logistic regression and the GBDTs for predicting micro- and macrovascular complications in individuals with prediabetes (Figure 2A) and diabetes (Figure 2B) over a 5‑year forecast horizon. For the prediabetes cohort, the mean AUROCs of the respective best model were in a range of 0.681 (SD 0.164) to 0.815 (SD 0.009). For the diabetes cohort, the mean AUROCs spanned over a range of 0.651 (SD 0.043) to 0.775 (SD 0.033). Nephropathy showed the best prediction performance in both cohorts. The prediction performance for macrovascular complications was generally better for individuals with prediabetes, whereas for microvascular complications (except nephropathy), the performance was better for individuals with diabetes. A comparison of the performance differences between logistic regression and GBDTs revealed that it depends on the cohort and the complication which model performs better. However, it can be observed that the prediction performance is comparable between the 2 models for most prediction tasks. Especially because in all cases the error bars are largely overlapping, we argue that no model should be preferred over the other. Additional performance metrics are reported in Multimedia Appendix 4. These metrics also show that both models performed comparably.

In Multimedia Appendix 5, we report the results of the calibration of the GBDTs. In summary, we observe that the GBDTs were already well calibrated before the post hoc calibration. However, in most cases, the calibration improved thereafter.

In Multimedia Appendix 7, we present the prediction performance for male and female individuals. No systematic deviation in performance was observed.

### Model Explainability

**Figure 3:**
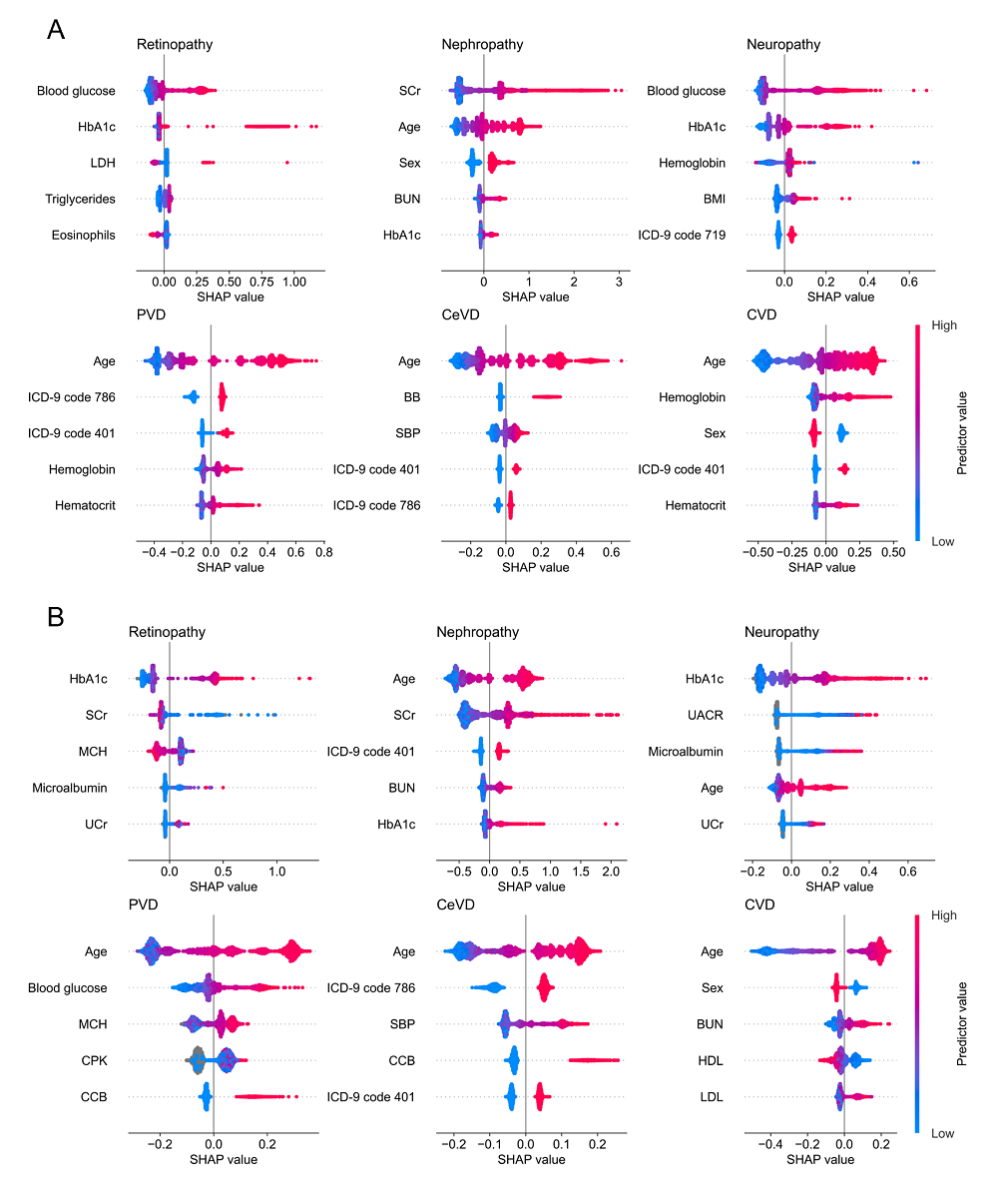
(A) SHAP plots for individuals with prediabetes. (B) SHAP plots for individuals with diabetes. For the SHAP plots, the ranking of the predictors is based on their importance listed in descending order. Each dot represents 1 individual, and its position on the x axis denotes its SHAP value. Elements with a positive (negative) SHAP value pull the prediction toward an increased (decreased) risk of developing a complication. The color of each dot is a representation of the corresponding predictor value, where red indicates a high, blue a low, and gray a missing value. BB: beta blocker; BUN: blood urea nitrogen; CCB: calcium channel blocker; CeVD: cerebrovascular disease; CPK: creatine phosphokinase; CVD: cardiovascular disease; HbA_1c_: glycated hemoglobin; HDL: high-density lipoprotein; ICD-9 719: other and unspecified disorders of joint; ICD-9 786: symptoms involving respiratory system and other chest symptoms; ICD-9 401: essential hypertension; LDH: lactate dehydrogenase; LDL: low-density lipoprotein; MCH: mean corpuscular hemoglobin; PVD: peripheral vascular disease; SBP: systolic blood pressure; SCr: serum creatinine; SHAP: Shapley additive explanations; UACR: albumin to creatinine ratio in urine; UCr: creatinine in urine.

Figures 3A and 3B show the 5 most important predictors for all 6 complications for individuals with prediabetes and diabetes, respectively. The HbA_1c_ value is an important predictor for all microvascular complications in both populations, where larger values are related to an increased risk of developing one of these complications. For nephropathy, increased age and large serum creatinine levels are the most important risk factors. In both populations, age is ranked as the most relevant predictor for all macrovascular complications. Hypertension (either determined by ICD‑9 code 401, elevated SBP, or a prescription of beta blockers or calcium channel blockers) is important for predicting PVD and CeVD in both populations. Male sex was identified as a risk factor for developing CVD.

### Robustness Checks

We experimented with other model variants to corroborate our findings. First, we tested sequential ML models (ie, recurrent neural networks with gated recurrent units and long short-term memory networks). Such models are hypothesized to improve prediction performance by taking into account the entire patient trajectory [37]. However, in our case, this did not improve the performance in comparison with our models. Second, we experimented with multitask learning where the different predictions are learned jointly. Again, our models were found to be superior. More details and the corresponding results can be found in Multimedia Appendix 9 [37].

## Discussion

### Principal Findings

We developed ML models to predict the risk of developing micro- or macrovascular complications in individuals with prediabetes or diabetes using routinely collected EHRs. Across all microvascular complications, the respective best ML model showed at least an acceptable performance for both cohorts. The only exception was retinopathy within the prediabetes cohort, where the performance was moderate. The reason for this might be the small number of individuals who developed retinopathy within the prediabetes cohort and a 5-year forecast horizon. The prediction performance for nephropathy in individuals with prediabetes showed good performance and, interestingly, thereby a better performance than the respective model for the diabetes cohort. It might be assumed that diabetes-related complications can be predicted with better performance in individuals with diabetes than in a population with prediabetes. This is because diabetes-related complications tend to occur earlier in individuals with diabetes because they have already passed through the stage of prediabetes. However, our diagnosis criteria for nephropathy (eGFR and albuminuria in addition to the ICD‑9 codes) might also include individuals whose nephropathy is not directly linked to prediabetes or diabetes. Furthermore, the prediabetes cohort contains almost 3 times more individuals than the diabetes cohort, which makes it easier for the ML models to learn the specific relationships leading to nephropathy.

For the macrovascular complications, we observed that the performance of the best model for PVD was acceptable in both cohorts. By contrast, for CeVD, the prediction performance was only moderate, which reduces its value for possible application in clinical settings. The best model for CVD for the prediabetes cohort showed a performance between moderate and acceptable (closer to acceptable), whereas for the diabetes cohort, it was slightly below this level. In addition, we observed that all macrovascular complications were easier to predict in individuals with prediabetes. One possible explanation would be that these complications are not as directly related to diabetes as are microvascular complications. The latter depend more on glycemic control (eg, blood glucose and HbA_1c_ levels), whereas macrovascular complications highly depend on additional risk factors (eg, age and blood pressure). In combination with the larger prediabetes cohort, this might be responsible for the better performance.

Furthermore, we observed that the comparative performance of the ML models depends upon the cohort and the specific complication. Overall, both ML models showed a similar prediction performance. Although GBDTs are widely considered to outperform logistic regression, a systematic review has found that a variety of ML models (including GBDTs) do not generally perform better than logistic regression in clinical prediction models [26]. However, in recent studies that used EHRs to build clinical prediction models, GBDTs significantly outperformed logistic regression [28,29]. Hence, it is not surprising that in this study, for some prediction tasks, logistic regression performed better, whereas for others, GBDTs performed better. In 1 case (retinopathy for individuals with diabetes), a large difference (>0.050) in the mean AUROC was observed between the 2 models. However, in this case, the number of outcomes compared with the overall sample size is small, thus resulting in large, overlapping error bars of the AUROCs. Hence, we argue that this difference does not reflect a substantial performance difference between the 2 models.

To identify the most important predictors of the GBDTs, SHAP values were calculated. These revealed that the HbA_1c_ value is an important predictor for all microvascular complications, and higher values are related to an increased risk. This relationship is well known and has been reported previously [4,38,39]. Serum creatinine is relevant for predicting nephropathy in individuals with prediabetes or diabetes. This finding is not surprising because serum creatinine is used to calculate the eGFR, which is one of the variables defining nephropathy. For macrovascular complications, age was identified as the most important predictor. This correlation is well known in the literature [40]. For PVD and CeVD, hypertension was related to an increased risk of developing one of these complications, which has been described previously [41,42].

### Comparison With Prior Work

To the best of our knowledge, no prediction models for micro- and macrovascular complications exist for individuals with prediabetes; hence, a comparison with prior work is not possible. By contrast, several prediction models for individuals with diabetes exist. Two previous studies have used Cox proportional hazards models [11,12]. Tanaka et al [11] built prediction models for coronary heart disease (CHD), stroke, noncardiovascular mortality, overt nephropathy, and retinopathy for a forecast horizon of 5 years. Our prediction models for stroke, nephropathy, and retinopathy outperformed their models; theirs performed better only for CHD and CVD (theirs: 0.725; ours: 0.686). The models by Basu et al [12] estimate the 10-year risk, which makes a direct comparison with our models with a 5-year forecast horizon difficult. In their work, the AUROCs on the internal validation set were often only moderate (ie, 0.550-0.680 for retinopathy, 0.600-0.840 for nephropathy, and 0.570-0.640 for neuropathy). In comparison, our best ML models achieved acceptable performances (ie, mean 0.726, SD 0.069; mean 0.775, SD 0.033; and mean 0.771, SD 0.031, respectively) on these complications for individuals with diabetes. Their model for myocardial infarction (MI) showed a performance similar to ours. For stroke, they reported an AUROC of 0.700 (our best model for CeVD: mean 0.651, SD 0.043).

ML models for predicting microvascular complications in individuals with diabetes were built in the study by Dagliati et al [13]. Therein, the authors reported acceptable performance for retinopathy and moderate performances for nephropathy and neuropathy (using logistic regression and a 5‑year forecast horizon). By contrast, our models showed acceptable performances for these 3 complications. Dworzynski et al [14] built ML models for cardiovascular disease, stroke, and chronic kidney disease with AUROCs of 0.690, 0.720, and 0.770, respectively. In comparison, we report mean AUROCs of 0.686 (SD 0.017), 0.651 (SD 0.043), and 0.775 (SD 0.033) for CVD, CeVD, and nephropathy, respectively. In a study by Ljubic et al [15], recurrent neural networks were used to estimate the 9-year risk for 10 different complications. Therein, separate performances for angina pectoris, ischemic CHD, and MI are reported, which are all grouped together in our outcome CVD. The performances of the models built by Ljubic et al [15] range from acceptable (MI) to good (ischemic CHD), thereby outperforming our model for CVD. Furthermore, their model for PVD outperformed ours (0.738-0.767 vs mean 0.715, SD 0.027, respectively). For nephropathy and neuropathy, our models performed better than theirs (nephropathy: mean 0.775, SD 0.033, vs 0.742-0.768, respectively; neuropathy: mean 0.771, SD 0.031, vs 0.715-0.746, respectively), whereas for retinopathy, their model outperformed ours by a small margin (0.728-0.796 vs mean 0.726, SD 0.069, respectively). Furthermore, a prediction model to estimate the 1‑year risk for chronic kidney disease was reported in the study by Song et al [17]. Therein, the authors state a prediction performance between acceptable and good (closer to good), which is slightly better than the performance of our model for nephropathy. ML models for predicting retinopathy and CVD within 3 years were reported in the study by Ravaut et al [18]. These models showed good performance for retinopathy (ours: acceptable) and acceptable performance for CVD (ours: moderate).

Overall, the prediction performance of our models for individuals with diabetes is comparable to the performances reported in prior work. However, the advantage of our study is that we are the first to also include prediction models for individuals with prediabetes. Prediction models for such a population are relevant because already half of the individuals when diagnosed with type 2 diabetes have had vascular complications [6]. Furthermore, our models are based on EHRs that include a large and representative population. This is because our EHRs contain data from multiple centers across Israel. In addition, our models account for an individual’s personal history by including a large number of predictors from demographics, biomarkers, medications, and comorbidities. In clinical settings, our prediction models could prove useful because they can be derived directly from EHRs and are therefore easily scalable. Furthermore, they allow for an early identification of individuals at risk. For these individuals, treatment could be administered earlier than usual and thus could increase the chances to prevent the complication. However, the prediction performance is in some cases worse than acceptable (AUROC <0.700). The benefit of these models—CeVD for both cohorts, retinopathy for the prediabetes cohort, and CVD for the diabetes cohort—is questionable.

### Limitations

This study has limitations. First, we used EHR data, which may be prone to wrongly reported or missing data. This may explain the small number of individuals who developed a specific complication within 5 years in comparison with individuals in data obtained from specialized diabetes clinics [13]. Moreover, it may also be the reason why our models did not achieve a better predictive performance although the general sample size was large. Furthermore, we only had access to data until 2013. It is possible that the reporting within the EHRs got better over time. Therefore, our analysis might be based on data from times when the quality of EHRs was lower than current standards. By contrast, an advantage of these EHRs is that they typically include measurements and diagnoses across the care continuum. This is due to their origin from a health insurance company. However, we cannot state this with absolute certainty because patients may switch among health care providers or receive treatment abroad. Finally, our EHRs did not contain information regarding living status (eg, income, diet, and physical activity) or sociodemographics (eg, race), which could be relevant predictors for estimating the risk of developing a micro- or macrovascular complication. Future studies may use more recent and more complete EHRs to improve the prediction performance. In addition, adjudicated claims or problem lists could be used to improve the reliability of the diagnosis of micro- and macrovascular complications.

Second, the data only covers an Israeli population. However, our approach could be generalized to other populations as well. This may be addressed in future work and, thereby, our ML models could additionally be validated on an external data set. As our EHRs did not contain information regarding ethnicity but encompass an Israeli population, we had to assume a non-Black ethnicity to calculate the eGFR. Furthermore, we could not assess whether our models perform equally well among different ethnicities.

Third, our definitions of prediabetes or diabetes were only based on HbA_1c_ measurements and recorded ICD-9 codes. We did not consider blood glucose measurements because the EHRs did not provide information on the time point of blood glucose measurement. Therefore, disease-defining fasting values were not available. However, this might also be beneficial because it ensures direct applicability of our ML models to EHRs, where the fasting state is not always recorded.

Fourth, our ML models were not trained on individuals at the time of diagnosis of prediabetes or diabetes but rather at a specific point in time (2008). The advantage of this approach is that it ensures an application of our ML models to all individuals with prediabetes or diabetes and not only to those who were just recently diagnosed. In addition, in clinical practice, the time point of prediabetes or diabetes diagnosis is often unknown, and the disease may have been already present for several years without being diagnosed.

Fifth, diagnosing nephropathy based on measurements of eGFR and ratio of albumin to creatinine in the urine may identify individuals whose nephropathy is unrelated or not exclusively related to prediabetes or diabetes but developed because of other reasons (eg, hypertension). Nonetheless, because prediabetes or diabetes is a major contributing factor for renal impairment, a correct prediction of nephropathy may be helpful, irrespective of its primary cause.

Sixth and last, we only consider a forecast horizon of 5 years. For the prediabetes cohort, a larger forecast horizon would be useful because diabetes-related complications typically occur later than in individuals with diabetes. Hence, an extension to larger forecast horizons would be an interesting analysis. However, it should be noted that the time span of our data set is not sufficient for such an analysis.

### Conclusions

Micro- and macrovascular complications are a major burden for individuals with prediabetes or diabetes. An early identification of individuals at risk is important because it could help to offer adequate treatment to prevent these complications. In this study, we built ML models to identify individuals with prediabetes or diabetes at high risk of developing micro- or macrovascular complications. For the first time, we showed that ML allows for predicting micro‑ and macrovascular complications in individuals with prediabetes. The prediction performance varied across complications and target populations but was acceptable for most prediction tasks.

## Appendix

Multimedia Appendix 1 Information on medications.

Multimedia Appendix 2 Predictors.

Multimedia Appendix 3 Hyperparameter tuning.

Multimedia Appendix 4 Performance metrics.

Multimedia Appendix 5 Model calibration.

Multimedia Appendix 6 Coefficients of logistic regression.

Multimedia Appendix 7 Risk group analysis.

Multimedia Appendix 8 Minimum Information About Clinical Artificial Intelligence Modeling (MI-CLAIM) checklist.

Multimedia Appendix 9 Robustness checks.

## Abbreviations

AUROC: area under the receiver operating characteristic curve
CeVD: cerebrovascular disease
CHD: coronary heart disease
CVD: cardiovascular disease
eGFR: estimated glomerular filtration rate
EHR: electronic health record
GBDT: gradient-boosted decision tree
HbA_1c_: glycated hemoglobin
ICD-9: International Classification of Diseases, Ninth Revision
MI: myocardial infarction
MI-CLAIM: Minimum Information About Clinical Artificial Intelligence Modeling
ML: machine learning
PVD: peripheral vascular disease
SBP: systolic blood pressure
SHAP: Shapley additive explanations
TRIPOD: Transparent Reporting of a Multivariable Prediction Model for Individual Prognosis or Diagnosis

## Data Availability

The data set analyzed in this study is not publicly available. To analyze the data, a data access agreement is needed.
