# Supplementary material for: Machine Learning for Predicting Micro- and Macrovascular Complications in Individuals With Prediabetes or Diabetes: Retrospective Cohort Study
Source: J Med Internet Res. 2023 Feb 27;25:e42181. doi: 10.2196/42181 (PMC10012007; doi:10.2196/42181)
Supplement: Multimedia Appendix 1 [file jmir_v25i1e42181_app1.docx]

**Multimedia Appendix 1. Information on medications**

**A List of antidiabetic medications**

The following antidiabetic medications were used as a diagnosis criterion for diabetes; these also include devices for self-measurement of blood glucose: Insulin, biguanides, sulfonylureas, DPP4-inhibitors, glinides, glitazones, GLP-1 receptor agonists, glucose meters, other medications (acrose, glucagen)

**B Classification of 50 most prescribed medications**

Statin: atorvastatin, lipitor, simvastatin-teva, simvastatin, stator

ACE-Inhibitor & ARB: ramipril, enaladex, tritace, cilaril, ramitens, enalapril, vascace, losardex

Diabetes medication: glucophage, glucomin

Other antihypertensive drug: disothiazide, cadex

Beta-blocker: normiten, normalol, cardiloc, lopresor

Calcium channel blocker: amlodipine, cartia, norvasc, vasodip

Proton-pump Inhibitor: omepradex, losec, lanton

Acetylsalicylic acid: micropirin, tevapirin

Vitamins, Minerals, Supplements: folic, supherb, tiptipot, optima

Anticoagulant: coumadin

Antidepressant: seroxat, cipramil, cipralex

Thyroid drug: eltroxin

Fibrate: bezafibrate, lipanor

Anxiolytic: lorivan, bondormin

Gout medication: alloril

Alpha-1 blocker: xatral

Bisphosphonate: fosalan, actonel

Asthma drug: seretide

Ezetimibe: ezetrol

Antihistamine: telfast
