# Supplementary material for: Machine Learning for Predicting Micro- and Macrovascular Complications in Individuals With Prediabetes or Diabetes: Retrospective Cohort Study
Source: J Med Internet Res. 2023 Feb 27;25:e42181. doi: 10.2196/42181 (PMC10012007; doi:10.2196/42181)
Supplement: Multimedia Appendix 2 [file jmir_v25i1e42181_app2.docx]

**Multimedia Appendix 2. Predictors**

Table A1. Included predictors for our ML models.

| Category | Predictors |
| --- | --- |
| Demographics | Age, BMI, Systolic blood pressure, Diastolic blood pressure |
| Biomarkers | RBC [10^6^/mcL], Hematocrit [%], Hemoglobin [g/100mL], MCH [pg/cell], MCV [fL], MCHC [g/dL], RDW [%], ESR [mm/h], WBC [10^3^/mcL], Lymphocytes [10^3^/mcL], Lymphocytes [%], Neutrophils [10^3^/mcL], Neutrophils [%], Monocytes [10^3^/mcL], Monocytes [%], Basophils [10^3^/mcL], Basophils [%], Eosinophils [10^3^/mcL], Eosinophils [%], Platelets [10^9^/L], MPV [fL], Glucose [mg/dL], HbA1c [%], Serum creatinine [mg/dL], BUN [mg/dL], Serum sodium [mmol/L], Serum potassium [mmol/L], Serum calcium [mg/dL], Phosphorus [mg/dL], ALT [IU/L], AST [IU/L], GGT [IU/L], ALP [IU/L], Bilirubin total [mg/dL], Uric acid [mg/100mL], CPK [IU/L], Cholesterol [mg/dL], HDL [md/dL], LDL [md/dL], Triglycerides [mg/dL], TSH [mcIU/mL], Total protein [g/dL], Serum albumin [g/dL], Iron [mcg/dL], Folate [mcg/L], Vitamin B12 [pg/mL], Urine [pH], SG, Urin protein [mg/dL], Urin leucocytes [1/mcL], PT [%], PT [s], Albumin/creatinine in urine [mg/g], Creatinine in urine [mg/dL], Microalbumin in urine [mg/dL], CRP [mg/L], INR, Free T4 [pmol/L], Erythrocytes, LDH [U/L] |
| Disease codes | 401 (Essential hypertension), 726 (Peripheral enthesopathies and allied syndromes), 799 (Other ill-defined and unknown causes of morbidity and mortality), 272 (Disorders of lipoid metabolism), 465 (Acute upper respiratory infections of multiple or unspecified sites), 724 (Other and unspecified disorders of back), 789 (Other symptoms involving abdomen and pelvis), 110 (Dermatophytosis), 780 (General symptoms), 786 (Symptoms involving respiratory system and other chest symptoms), 784 (Symptoms involving head and neck), 692 (Contact dermatitis and other eczema), 79 (Viral infection in conditions classified elsewhere and of unspecified site), 278 (Overweight, obesity and other hyperalimentation), 719 (Other and unspecified disorders of joint), 466 (Acute bronchitis and bronchiolitis), 729 (Other disorders of soft tissues), 790 (Nonspecific findings on examination of blood), 367 (Disorders of refraction and accommodation), 372 (Disorders of conjunctiva) |
| Medications | Statin, ACE-Inhibitor & ARB, Diabetes medication, Other antihypertensive drug, Beta-blocker, Calcium channel blocker, Proton-pump Inhibitor, Nonsteroidal anti-inflammatory drug, Vitamins, Minerals, Supplements, Anticoagulant, Antidepressant, Thyroid drug, Fibrate, Anxiolytic, Gout medication, Alpha-1 blocker, Bisphosphonate, Asthma drug, Cholesterol absorption inhibitor, Antihistamine |

Abbreviations: RBC: red blood cell count, MCH: mean corpuscular hemoglobin, MCV: mean corpuscular volume, MCHC: mean corpuscular hemoglobin concentration, RDW: red cell distribution width, ESR: erythrocyte sedimentation rate, WBC: white blood cell count, MPV: mean platelet volume, BUN: blood urea nitrogen, ALT: alanine aminotransferase, AST: aspartate aminotransferase, GGT: gamma-glutamyl transferase, ALP: alkaline phosphatase, CPK: creatine phosphokinase, HDL: high‑density lipoprotein, LDL: low‑density lipoprotein, TSH: thyroid-stimulating hormone, SG: urinary specific gravity, PT: prothrombin time, CRP: C‑reactive protein, INR: international normalized ratio, LDH: lactate dehydrogenase.
