# Supplementary material for: Machine Learning for Predicting Micro- and Macrovascular Complications in Individuals With Prediabetes or Diabetes: Retrospective Cohort Study
Source: J Med Internet Res. 2023 Feb 27;25:e42181. doi: 10.2196/42181 (PMC10012007; doi:10.2196/42181)
Supplement: Multimedia Appendix 3 [file jmir_v25i1e42181_app3.docx]

**Multimedia Appendix 3. Hyperparameter tuning**

To improve our ML models, we conducted a randomized search with 20 iterations over the following hyperparameter spaces:

Logistic regression:

- C: [10^-3^, 10^3^]

GBDTs:

- iterations: [50, 500)
- learning_rate: [10^-3^, 1]
- depth: [3, 7]

RNN with gated recurrent units:

- n_cells: {3, 5, 8, 10, 15, 50}
- dropout_rate: {0, 0.2, 0.5}
- recurrent_dropout_rate: {0, 0.2, 0.5}
- learning_rate: {0.0001, 0.001, 0.01, 0.1}
- batch_size: {64, 128}
- epochs: [5, 30)
