# Supplementary material for: Machine Learning for Predicting Micro- and Macrovascular Complications in Individuals With Prediabetes or Diabetes: Retrospective Cohort Study
Source: J Med Internet Res. 2023 Feb 27;25:e42181. doi: 10.2196/42181 (PMC10012007; doi:10.2196/42181)
Supplement: Multimedia Appendix 4 [file jmir_v25i1e42181_app4.docx]

**Multimedia Appendix 4. Performance metrics**

In Table A2, we report the area under the receiver operating characteristic (AUROC), area under the precision recall curve (AUPRC), sensitivity, specificity, and balanced accuracy for both cohorts, both ML models, and all outcomes. For the sensitivity, specificity, and balanced accuracy, we chose the threshold that corresponds to the point on the receiver operating characteristic closest to a perfect classifier. We report the mean (SD) across the five different test sets.

Table A2: Performance metrics.

| Metric | Population | Outcome Model | Retino-pathy | Nephro-pathy | Neuro-pathy | PVD | CeVD | CVD |
| --- | --- | --- | --- | --- | --- | --- | --- | --- |
| AUROC | Prediabetes | Logistic regression | 0.657 (0.106) | 0.807 (0.013) | 0.727 (0.070) | 0.730 (0.047) | 0.687 (0.009) | 0.707 (0.007) |
|  | Prediabetes | GBDTs | 0.681 (0.164) | 0.815 (0.009) | 0.706 (0.065) | 0.727 (0.047) | 0.693 (0.013) | 0.705 (0.017) |
|  | Diabetes | Logistic regression | 0.673 (0.078) | 0.763 (0.037) | 0.745 (0.030) | 0.698 (0.042) | 0.651 (0.043) | 0.686 (0.017) |
|  | Diabetes | GBDTs | 0.726 (0.069) | 0.775 (0.033) | 0.771 (0.031) | 0.715 (0.027) | 0.646 (0.046) | 0.680 (0.050) |
| AUPRC | Prediabetes | Logistic regression | 0.006 (0.002) | 0.275 (0.016) | 0.014 (0.006) | 0.052 (0.019) | 0.072 (0.005) | 0.099 (0.017) |
|  | Prediabetes | GBDTs | 0.009 (0.003) | 0.290 (0.013) | 0.009 (0.002) | 0.052 (0.018) | 0.070 (0.008) | 0.096 (0.008) |
|  | Diabetes | Logistic regression | 0.036 (0.013) | 0.296 (0.063) | 0.126 (0.055) | 0.062 (0.015) | 0.087 (0.018) | 0.118 (0.025) |
|  | Diabetes | GBDTs | 0.031 (0.008) | 0.295 (0.037) | 0.110 (0.049) | 0.081 (0.022) | 0.093 (0.024) | 0.117 (0.032) |
| Sensitivity | Prediabetes | Logistic regression | 0.714 (0.239) | 0.738 (0.060) | 0.721 (0.121) | 0.752 (0.041) | 0.678 (0.152) | 0.713 (0.067) |
|  | Prediabetes | GBDTs | 0.743 (0.277) | 0.748 (0.069) | 0.756 (0.091) | 0.733 (0.093) | 0.773 (0.043) | 0.807 (0.095) |
|  | Diabetes | Logistic regression | 0.795 (0.173) | 0.768 (0.081) | 0.677 (0.116) | 0.756 (0.109) | 0.590 (0.097) | 0.807 (0.125) |
|  | Diabetes | GBDTs | 0.824 (0.199) | 0.824 (0.077) | 0.792 (0.058) | 0.854 (0.126) | 0.692 (0.176) | 0.777 (0.085) |
| Specificity | Prediabetes | Logistic regression | 0.687 (0.152) | 0.748 (0.058) | 0.716 (0.078) | 0.640 (0.017) | 0.634 (0.133) | 0.620 (0.063) |
|  | Prediabetes | GBDTs | 0.687 (0.201) | 0.767 (0.058) | 0.648 (0.067) | 0.651 (0.106) | 0.556 (0.043) | 0.518 (0.128) |
|  | Diabetes | Logistic regression | 0.591 (0.210) | 0.665 (0.055) | 0.747 (0.120) | 0.589 (0.090) | 0.697 (0.087) | 0.519 (0.120) |
|  | Diabetes | GBDTs | 0.637 (0.121) | 0.635 (0.072) | 0.682 (0.056) | 0.508 (0.117) | 0.552 (0.164) | 0.565 (0.113) |
| Balanced accuracy | Prediabetes | Logistic regression | 0.629 (0.089) | 0.740 (0.008) | 0.675 (0.053) | 0.686 (0.027) | 0.651 (0.014) | 0.662 (0.010) |
|  | Prediabetes | GBDTs | 0.643 (0.121) | 0.754 (0.007) | 0.659 (0.054) | 0.682 (0.038) | 0.659 (0.010) | 0.658 (0.019) |
|  | Diabetes | Logistic regression | 0.651 (0.062) | 0.710 (0.034) | 0.693 (0.025) | 0.652 (0.047) | 0.631 (0.032) | 0.652 (0.007) |
|  | Diabetes | GBDTs | 0.688 (0.067) | 0.723 (0.030) | 0.718 (0.026) | 0.661 (0.026) | 0.609 (0.030) | 0.660 (0.029) |
