# Supplementary material for: Machine Learning for Predicting Micro- and Macrovascular Complications in Individuals With Prediabetes or Diabetes: Retrospective Cohort Study
Source: J Med Internet Res. 2023 Feb 27;25:e42181. doi: 10.2196/42181 (PMC10012007; doi:10.2196/42181)
Supplement: Multimedia Appendix 5 [file jmir_v25i1e42181_app5.docx]

**Multimedia Appendix 5. Model calibration**

**
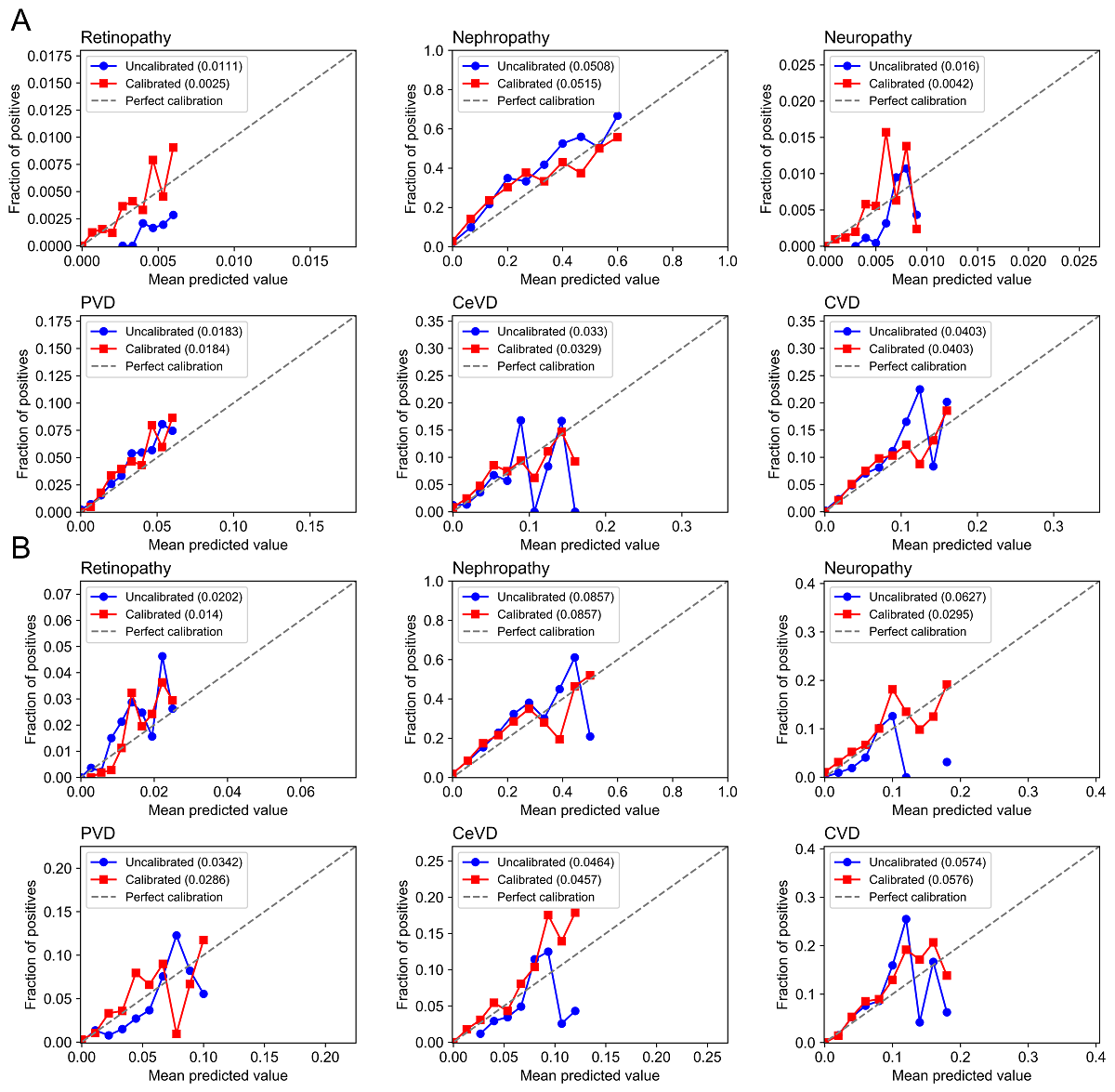
**

Figure A1: Calibration plots for the GBDTs. The Brier score before and after model calibration is reported in parentheses in the legend. (A) Prediabetes cohort. (B) Diabetes cohort.
