# Supplementary material for: Machine Learning for Predicting Micro- and Macrovascular Complications in Individuals With Prediabetes or Diabetes: Retrospective Cohort Study
Source: J Med Internet Res. 2023 Feb 27;25:e42181. doi: 10.2196/42181 (PMC10012007; doi:10.2196/42181)
Supplement: Multimedia Appendix 6 [file jmir_v25i1e42181_app6.docx]

**Multimedia Appendix 6. Coefficients of logistic regression**


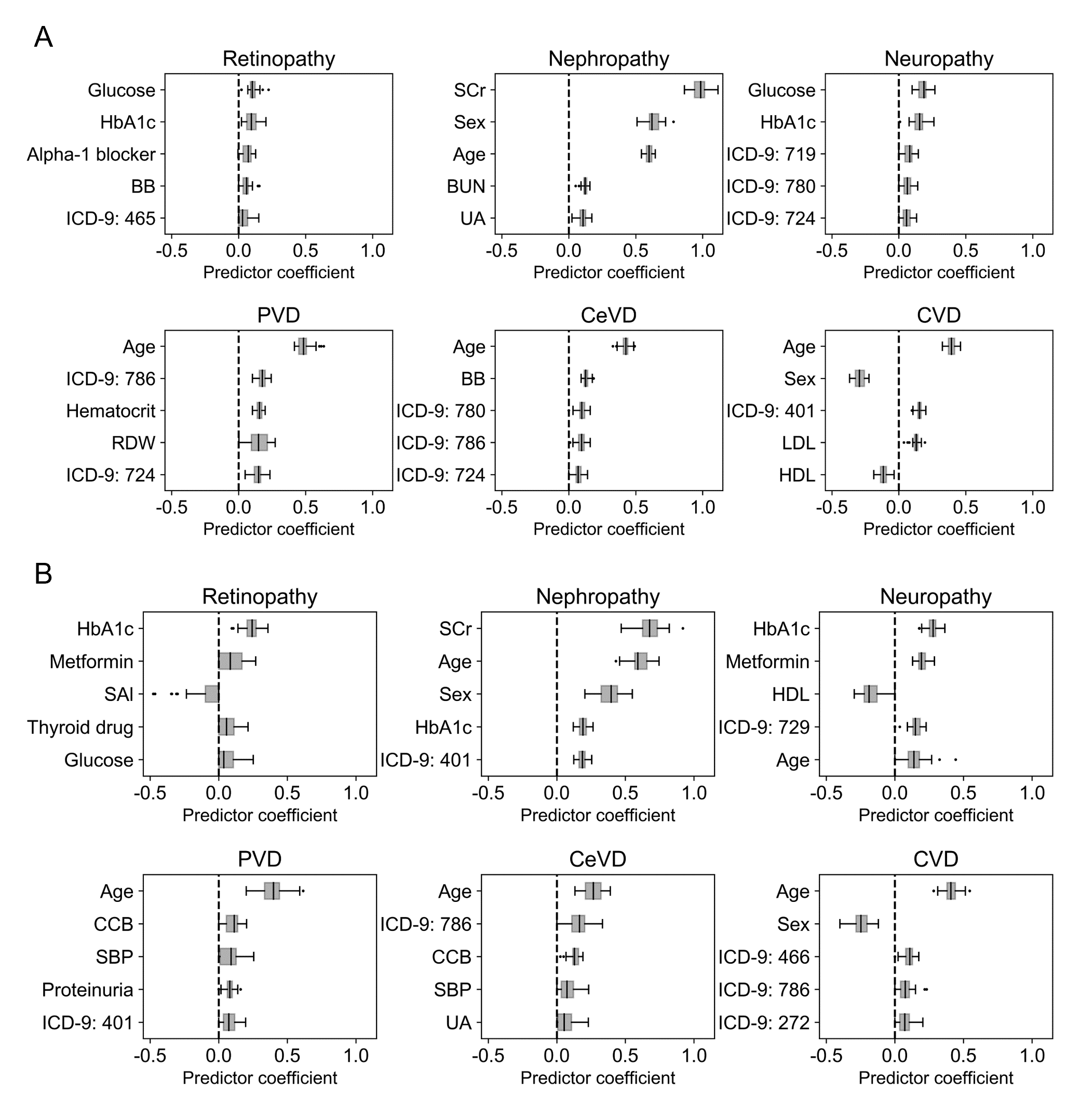


Figure A2: Coefficients of the logistic regression of the five most important predictors for each outcome and both cohorts. (A) Prediabetes cohort. (B) Diabetes cohort.

Abbreviations: BB: Beta blocker, ICD‑9 465: Acute upper respiratory infections of multiple or unspecified sites; SCr: Serum creatinine; BUN: Blood urea nitrogen; UA: Uric acid; ICD‑9 719: Other and unspecified disorders of joint; ICD‑9 780: General symptoms; ICD‑9 724: Other and unspecified disorders of back; ICD‑9 786: Symptoms involving respiratory system and other chest symptoms; RDW: Red cell distribution width; ICD‑9 401: Essential hypertension; LDL: Low density lipoprotein; HDL: High density lipoprotein; SAl: Serum albumin; ICD‑9 729: Other disorders of soft tissues; CCB: Calcium channel blocker; SBP: Systolic blood pressure; ICD‑9 466: Acute bronchitis and bronchiolitis; ICD‑9 272: Disorders of lipoid metabolism.
