# Supplementary material for: Machine Learning for Predicting Micro- and Macrovascular Complications in Individuals With Prediabetes or Diabetes: Retrospective Cohort Study
Source: J Med Internet Res. 2023 Feb 27;25:e42181. doi: 10.2196/42181 (PMC10012007; doi:10.2196/42181)
Supplement: Multimedia Appendix 7 [file jmir_v25i1e42181_app7.docx]

**Multimedia Appendix 7. Risk group analysis**


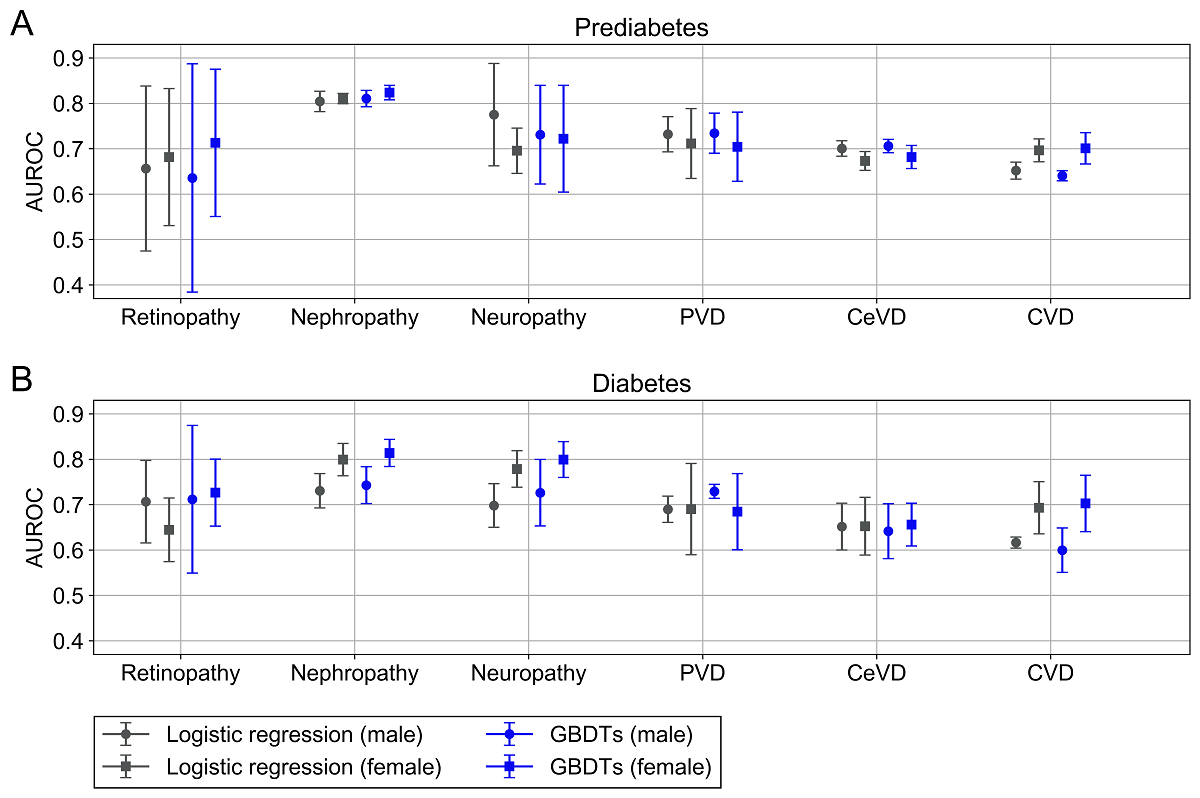


Figure A3: Comparison of the prediction performance for male versus female individuals. We report the mean of the AUROC across the five different test sets. The error bars denote ±SD. (A) Prediabetes cohort. (B) Diabetes cohort.
