# Supplementary material for: Machine Learning for Predicting Micro- and Macrovascular Complications in Individuals With Prediabetes or Diabetes: Retrospective Cohort Study
Source: J Med Internet Res. 2023 Feb 27;25:e42181. doi: 10.2196/42181 (PMC10012007; doi:10.2196/42181)
Supplement: Multimedia Appendix 8 [file jmir_v25i1e42181_app8.docx]

**Multimedia Appendix 8. MI-CLAIM checklist**

Table A3: MI-CLAIM checklist [36].

| **Study design (Part 1)** | **Completed** | **Notes** |
| --- | --- | --- |
| The clinical problem in which the model will be employed is clearly detailed in the paper. | X (Introduction) | To predict micro- and macrovascular complications in individuals with (pre) diabetes. |
| The research question is clearly stated. | X (Introduction) | Can ML predict micro- and macrovascular complications in individuals with (pre) diabetes? Does logistic regression or gradient boosted decision trees work better? |
| The characteristics of the cohorts (training and test sets) are detailed in the text. | n/a | We apply 5-fold cross-validation, therefore we do not have separate train and test cohorts. The characteristics of the entire cohorts are presented in Table 1 (prediabetes) and Table 2 (diabetes). |
| The cohorts (training and test sets) are shown to be representative of real-world clinical settings. | X (Methods) | We utilized EHRs, which are often routinely collected in real-world clinical practice. |
| The state-of-the-art solution used as a baseline for comparison has been identified and detailed. | n/a | No clinical state‑of‑the‑art solutions exist for predicting micro- and macrovascular complications in individuals with (pre) diabetes. Hence, we compared two different ML models. |
| **Data and optimization (Parts 2, 3)** | **Completed** | **Notes** |
| The origin of the data is described and the original format is detailed in the paper. | X (Methods) | Origin of the data (an Israeli health provider) is described, but original format is not detailed in the paper. |
| Transformations of the data before it is applied to the proposed model are described. | X (Methods) |  |
| The independence between training and test sets has been proven in the paper. | X (Methods) | We use 5-fold cross-validation, therefore, training and test sets are independent. |
| Details on the models that were evaluated and the code developed to select the best model  are provided. | X (Methods and Multimedia Appendix 3) |  |
| Is the input data type structured or unstructured? | structured |  |
| **Model performance (Part 4)** | **Completed** | **Notes** |
| The primary metric selected to evaluate algorithm performance (e.g., AUC, F-score, etc.), including the justification for selection, has been clearly stated. | X (Methods) | Area under the receiver operating curve. |
| The primary metric selected to evaluate the clinical utility of the model (e.g., PPV, NNT, etc.), including the justification for selection, has been clearly stated. | X (Multimedia Appendix 4) | Area under precision recall curve, sensitivity, specificity, and balanced accuracy. |
| The performance comparison between baseline and proposed model is presented with the appropriate statistical significance. | n/a | We compare the performance of two ML models, namely logistic regression and GDBTs, and report the statistical significance. |
| **Model examination (Part 5)** | **Completed** | **Notes** |
| Examination technique 1 | X (Methods, Results) | Shapley Additive Explanations (SHAP) summary plots to show the impact of a predictor on the model performance as a function of the predictor value. |
| Examination technique 2 | X (Multimedia Appendix 6) | Coefficients of the logistic regression. |
| A discussion of the relevance of the examination results with respect to model/algorithm performance is presented. | X (Discussion) |  |
| A discussion of the feasibility and significance of model interpretability at the case level if examination methods are uninterpretable is presented. | n/a | The logistic regression is interpretable at the case level. |
| A discussion of the reliability and robustness of the model as the underlying data distribution shifts is included. | n/a | A longitudinal panel dataset was used. Future work may need to recalibrate models (e.g., annually) as new data becomes available. |
| **Reproducibility (Part 6): choose appropriate tier of transparency** | **Completed** | **Notes** |
| Tier 1: complete sharing of the code | X |  |
| Tier 2: allow a third party to evaluate the code for accuracy/fairness; share the results of this evaluation |  |  |
| Tier 3: release of a virtual machine (binary) for running the code on new data without sharing its details |  |  |
| Tier 4: no sharing |  |  |
