# Supplementary material for: Machine Learning for Predicting Micro- and Macrovascular Complications in Individuals With Prediabetes or Diabetes: Retrospective Cohort Study
Source: J Med Internet Res. 2023 Feb 27;25:e42181. doi: 10.2196/42181 (PMC10012007; doi:10.2196/42181)
Supplement: Multimedia Appendix 9 [file jmir_v25i1e42181_app9.docx]

**Multimedia Appendix 9. Robustness checks**

We performed two additional analyses to check whether alternative methods in predicting micro‑ and macrovascular complications in individuals with (pre) diabetes might lead to better predictive performance.

*Sequential machine learning models*

First, we made use of sequential machine learnings models. This is motivated by recent research suggesting that modeling patient trajectories may encode valuable information for prediction [37]. The underlying idea is that learning temporal dynamics in important risk factors or other variables such as HbA_1c_ or serum creatinine could increase the overall prediction performance. For example, increasing levels of HbA_1c_ over time may indicate a trajectory towards microvascular complications, whereas constant values may not.

To this end, we now use the entire patient trajectories (data from 2003 until baseline, i.e., 2008). We further experimented with two different modeling approaches. (1) We performed feature engineering and used eight summary statistics to model the trajectories (minimum, maximum, mean, standard deviation, number of measurements, difference between maximum and minimum, difference between last and first measurement, difference between measurements at baseline and two years before). Since taking eight summary statistics of 63 trajectory predictors would increase the number of predictors in comparison to the outcomes drastically and may lead to overfitting, we only considered six basic trajectory predictors (BMI, SBP, DBP, HbA_1c_, glucose, and serum creatinine) in addition to age and sex. We again used GBDTs as the underlying machine learning model. We refer to this as “modeled trajectory” in the following. (2) We used recurrent neural networks (RNNs) with gated recurrent units (GRUs), which can take the entire patient trajectory as input. We also experimented with long-short term memory (LSTM) networks; however, these were inferior (not shown later, for brevity).

Figure A4 shows the performance of (1) our “standard method” (GBDTs with only the last value of the trajectory as input, from the main paper), (2) the “modeled trajectory” via feature engineering, and (3) the RNN with GRUs. For all three methods, we used the same eight predictors. First, we see that the sequential neural network is inferior. The “modeled trajectory” via feature engineering is on par with the “standard method”. In sum, the results demonstrate the effectiveness of the “standard method” from the main paper.


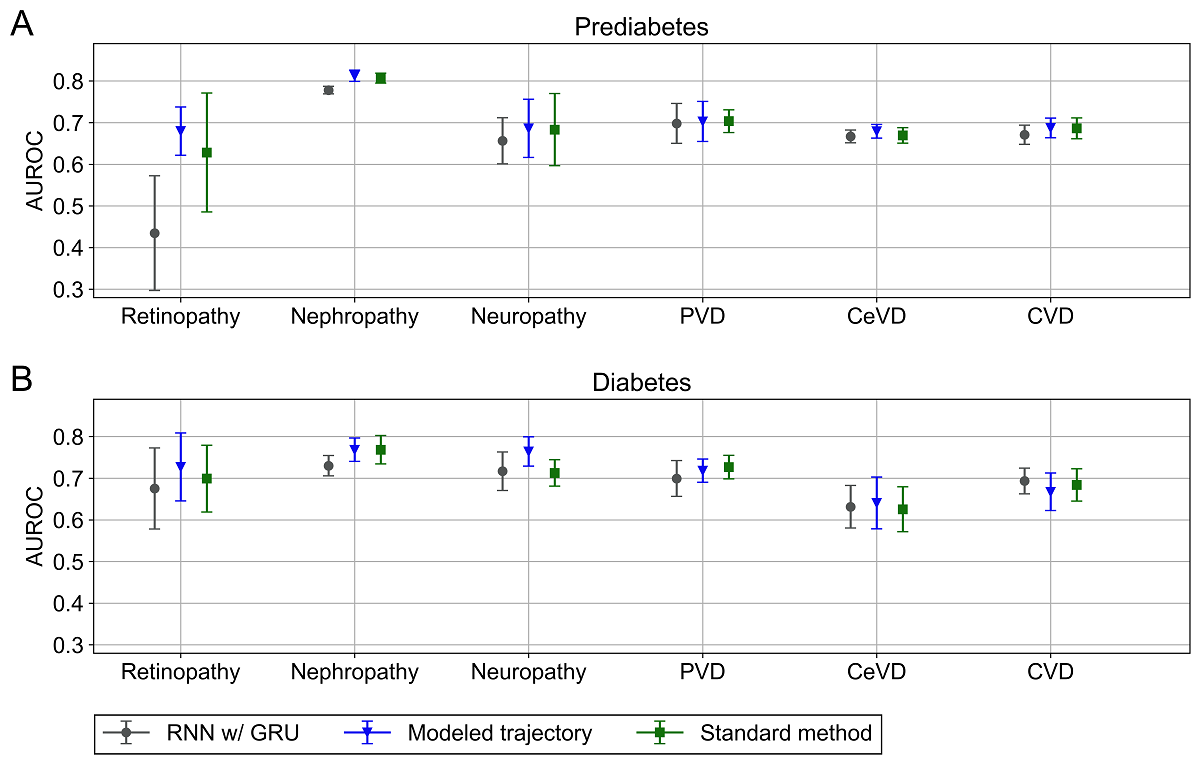


Figure A4: Performance of the standard method, the modeled trajectory via summary statistics, and the RNN with GRUs for individuals with prediabetes (A) and diabetes (B). We report the mean of the AUROC across the five different test sets. The error bars denote ±SD.

*Multi-task learning*

We applied a simple multitask learning approach, where we predicted all six complications using a single classifier. Here, the idea is that learning all complications jointly may detect certain underlying patterns in individuals with an overall deteriorating health and thereby increasing the predictive performance.

We implemented two variants. (1) We used the multi-task procedure described above and train the machine learning model to predict all outcomes and the additional label “any complication” jointly. However, we had to use as a baseline cohort only patients that had no history of any of the six complications at baseline. (2) We included a second multitask approach, where a single classifier for each complication was built but with the additional label of “any complication”. For both variants, we again use GBDTs.

Figure A5 shows the performance of the standard method, the multitask learning variant (1), and the multitask learning variant (2). For all three methods, we used the same baseline cohort, where individuals with any complication before or at baseline were excluded. None of the multi-task methods showed consistent improvements in the prediction performance, thereby confirming the effectiveness of our machine learning method from the main paper.


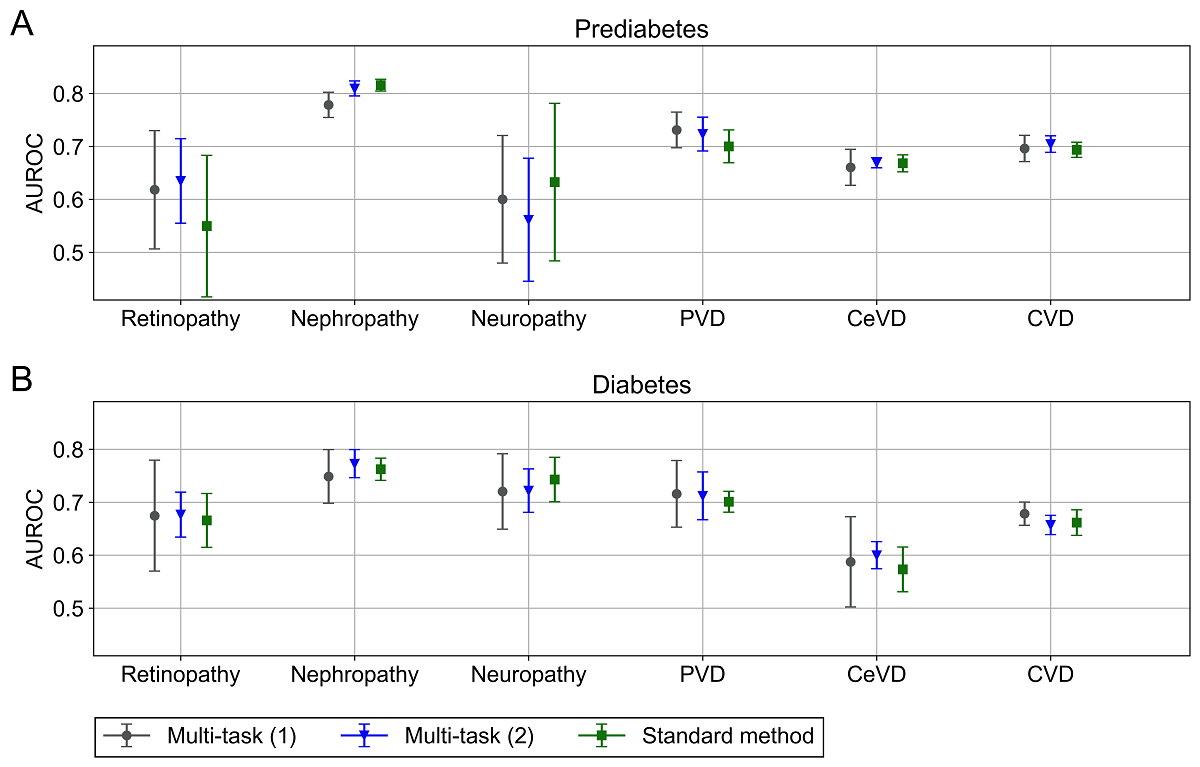


Figure A5: Performance of the standard method and two different variants for multi‑task learning in individuals with prediabetes (A) and diabetes (B). We report the mean of the AUROC across the five different test sets. The error bars denote ±SD.
